# Supplementary material for: Prevalence and factors which influence early antenatal booking among women of reproductive age in Tanzania: An analysis of data from the 2015-16 Tanzania Demographic Health Survey and Malaria Indicators Survey
Source: PLoS One. 2021 Apr 6;16(4):e0249337. doi: 10.1371/journal.pone.0249337 (PMC8023495; doi:10.1371/journal.pone.0249337)
Supplement: Attachment — Submitted filename: Response to Reviewers..doc [file pone.0249337.s001.doc]

**RESPONSE TO REVIEWERS' COMMENTS**

**Editor’s comments**

**Comment 1**

Please ensure that your manuscript meets PLOS ONE's style requirements, including those for file naming. The PLOS ONE style templates can be found at <https://journals.plos.org/plosone/s/file?id=wjVg/PLOSOne_formatting_sample_main_body.pdf> and <https://journals.plos.org/plosone/s/file?id=ba62/PLOSOne_formatting_sample_title_authors_affiliations.pdf>

**Response 1**

*The manuscript meets PLOS ONE style*

**Comment 2**

In your ethics statement in the Methods section and in the online submission form, please clarify whether all data were fully anonymized before you accessed them.

In addition, PLOS ONE requires statistical methods to be described in enough detail to allow suitably skilled investigators to fully replicate and evaluate a study. After internal review, we were concerned that the experimental and statistical methods may not detailed enough to meet these criteria.

Please revise your manuscript to include a more detailed description of your statistical methods.

***Response 2***

*Data were extracted from the original file where information which were not a focus of the study were dropped. Also, the dataset was cleaned by removing incomplete cases; line 191 to 193*

*Method section is revised and description of data analysis is included line 205 to 208*

**Comment 3**

Your ethics statement should only appear in the Methods section of your manuscript. If your ethics statement is written in any section besides the Methods, please move it to the Methods section and delete it from any other section. Please ensure that your ethics statement is included in your manuscript, as the ethics statement entered into the online submission form will not be published alongside your manuscript.

**Response 3**

*Author agree with the comment and ethical statement is moved to the method section*

**Comment 4**

Thank you for stating in the text of your manuscript "Data collection and the survey content and protocol were approved by Tanzania’s National Institute for Medical Research (NIMR), the Zanzibar Medical Ethics and Research Committee (ZAMREC), the Institutional Review Board of ICF International, and the Centers for Disease Control and Prevention in Atlanta, USA. Participants provided verbal consents and the household interviews took place at the private condition. Participants who were under 18years old written consent were requested from their parents or guardians."

Please describe how verbal consent was documented and witnessed, and why written consent was not obtained for these participants.

Please also clarify whether written informed consent was obtained (not just requested) from parents of participants under 18 years of age.

Please add all of this information to your manuscript text and the ethics statement in the online submission form.

We look forward to hearing from you.

**Response 4**

*Author changed the ethical statement to suit the nature of the study a copy is also stated here*

*This study used secondary data without involving any human subjects which therefore did not require formal ethical approval. On the other hand, the request to use the data was sought from DHS measures. Permission was given subject to using the data for this particular research topic only and publishing the findings in a peer-reviewed journal.*

**Comment 5**

Thank you for providing a Data Availability Statement and indicating that the data were retrieved from the DHS. In order for your statement to meet our data sharing policy requirements (https://journals.plos.org/plosone/s/data-availability), however, we ask that you provide the name of the data set obtained from the DHS, to allow other researchers to request the minimal data set

**Response 6**

*This needs registration, After registration download datasets, list of datasets arranged according to countries will open, then find Tanzania in the list; available datasets for Tanzania are listed and select Tanzania 2015-16 data set, open it. Again the list of files are listed, select individual recorde file TZIR7BSV.ZIP, open the zip file and then the dataset used in this study is there.*

*It is that I am not allowed to share the dataset, MEASURE DHS are in-charge of the data, they provide upon request.*

*The link to registration is provided below*

[*https://dhsprogram.com/data/dataset_admin/login_main.cfm?CFID=1243545&CFTOKEN=6d7e7d96a77670b0-90440413-E03C-C102-ABBC22025A1CD7FA*](https://dhsprogram.com/data/dataset_admin/login_main.cfm?CFID=1243545&CFTOKEN=6d7e7d96a77670b0-90440413-E03C-C102-ABBC22025A1CD7FA)

*This needs registration, After registration download datasets, list of datasets arranged according to countries will open, then find Tanzania in the list; available datasets for Tanzania are listed and select Tanzania 2015-16 data set, open it. Again the list of files are listed, select individual recorde file TZIR7BSV.ZIP, open the zip file and then the dataset used in this study is there.*

*It is that I am not allowed to share the dataset, MEASURE DHS are in-charge of the data, they provide upon request.*

**Reviewers' comments:**

**Reviewer 1**

Comment 1

In the Introduction, the author needs to fully and thoroughly review previous research regarding antenatal care (ANC) visits. There are several review articles related to positive and negative factors of ANC participation. In both high and low-middle income countries, low maternal education, non-marital status, and high parity are associated with inadequate use of ANC (Simkhada, 2008; Feijen-de Jong, 2012). Then, the author needs to describe why the study on Tanzania is required at this time.

***Response 1***

*The introduction is reviewed and the suggested literature were visited and used in the introduction Line 76 to line 135*

Comment 2

The reviewer does not clearly make the connection between the last sentence “little is known …”, in the last paragraph and the second to last paragraph in the Introduction. Also, there is no statement of the objectives of the study.

***Response 2***

*The two paragraphs are re-written and the line of argument is connected*

**Comment 3**

The author stated that the WHO recommended antenatal model including numbers of ANC visit in 2016. However, the author uses data collected in 2015-2016. The reviewer assumes that ANC provision may have followed the previous WHO recommendation at the time of data collection in Tanzania. It may be reasonable to evaluate data in 2015-2016 according to the previous WHO recommendation and the national guideline of ANC at the time of data collection. In addition, more detail of ANC related conditions in Tanzania, which are distribution of healthcare personnel and healthcare facilities, and quantity and quality of ANC provision, will be informative for readers of this manuscript, if the author adds them in the Introduction section.

***Response 3***

*Author agrees with the comment and the WHO recommendation presented in the introduction is the one used during the time of survey line 84 to line 97*

**Comment 4**

If the author provided an association between religion and ANC visits, it would be informative, because early ANC visits were less likely to be observed in Zanzibar than mainland, where there are more Muslims.

**Response 4**

*Author agree with the observation and explanation on to why there are low proportion of early booking among pregnant women in Zanzibar Islands is provided, line 337 to line 344*

**Comment 5**

If the author considered confounders and/or mediators among independent variables, it would be informative to the interpretation of the findings.

***Response 5***

**Comment 6**

The author mentioned that teenage pregnant women are less likely to initiate ANC visit than the older pregnant women. Is there a possibility of unplanned teenage pregnancy without marriage? How about conditions for and provision of youth friendly services in Tanzania? How about cultural and/or social norms regarding teenage pregnancy? The reviewer considers marital status, cultural and/or social norms, and access to and quality of youth friendly services provision are important factors when evaluating maternity services including ANC and consequences/outcomes among teenage pregnancy.

***Response 6***

*The reasons for teenage pregnant women to initiate ANC clinics late is added in line 302 to line 307. Marital status was among the variables which were considered to influence early antenatal booking. The variable did not show significant relationship with ANC booking in the cross tabulation and then it was not taken to regression analysis.*

**Comment 7**

In the last paragraph of the Discussion section, the author should discuss in-depth the Zanzibar conditions from the perspective of religious, cultural and/or social norms, distribution of healthcare personnel, and quantity and quality of ANC service provision.

***Response 7***

*The message is included in the discussion*

**Comment 8**

It would be better to describe the limitations of this study in the Discussion section.

***Response 8***

*A paragraph on limitation of the study is added line 363-368*

**Comment 9**

Abstract: In the Results section, AOR and CI should be spelled out when used for the first time.

**Response 9**

*The two abbreviations are now spelled out in the first time used in the abstract line 36.*

**Comment 10**

Results section: In the “Factors which influence early antenatal booking”, AOR and CI should be spelled out when used at the first time.

***Response 10***

*The two abbreviations are now spelled out in the first time used in the results section line 248.*

**Comment 11**

Discussion section: The last sentence of the third to the last paragraph needs referencing. ‘Previous studies have reported that pregnant women ….. if compared with those without complication.’

***Response 11***

***The sentence is re-phrased line 334***

**Comment 12**

Overall, the reviewer recommends basic editing of the article, such as adjusting spacing between word and word, word and brackets, and after comma, because there are unnecessary spaces and/or no spaces.

***Response 12***

***Authors agree with the recommendation and the editing was done***

**Reviewer 2**

**Comment 1**

Abstract: Minor grammatical errors to be corrected

**Response 1**

*The grammatical errors are corrected*

**Comment 2**

Introduction: Requires cleaning up. The literature review in the introduction section needs to be summarized especially the area on WHO 2016 report. It is more of copy and paste.

**Response 2**

*The introduction is re-written and paraphrasing of reference documents used is done*

**Comment 3**

Methods

i) Study population: Conflicting figures from the previous statement

**Response**

*The figure is corrected*

ii) Sampling technique: The sampling technique is faulty. What you did is a multi-stage sampling and not two stages. Tanzania was divided into 8 zones. This should have formed your clusters or strata from where you will select the households. How did you arrive at 608 clusters?
    How you reached your study participants after selecting the households was not stated

**Response**

*The study used secondary data. The method section has been published in the report of Tanzania Demographic and Health Survey and Malaria Indicator Survey 2015-2016*

iii) Data collection tool: Was the translated questionnaire to Kiswahili Tanzania’s national language validated? If not it is a flaw in this study
     How you administered the questionnaire to the study participants was not expla9ined

**Response**

*The study used secondary data. The method section has been published in the report of Tanzania Demographic and Health Survey and Malaria Indicator Survey 2015-2016*

iv) Data analysis: Recast the first sentence

**Response**

*The first sentence is re-casted*

**Comment 4**

Results: What happened to 117 of the study participants?

**Response 4**

*Study participants with missing information on timing for ANC visits were removed*

**Comment 5**

Discussions: Requires cleaning up for grammatical errors

**Response 5**

*The grammatic errors corrected*

**Comment 6**

Conclusion: What you did is not an interventional study so delete

**Response 6**

*It is true what I did is not an intervention study but, in the conclusion, author is recommending for an intervention study which is more conclusive than a cross-sectional survey.*

**Comment 7**

Declarations: The questionnaire was used as interview guide to interview your study participants.
**Response 7**

*Yes, questionnaire was used to gather data but the author used the already developed data set*

**Comment 8**

References: Revisit to be consistent,

**Response 8**

*The list of references is revisited*
